# Thermovibrationally Driven Ring-Shaped Particle Accumulations in Corner-Heated Cavities with the *D_2_h* Symmetry

**DOI:** 10.3390/mi17010039

**Published:** 2025-12-29

**Authors:** Balagopal Manayil Santhosh, Marcello Lappa

**Affiliations:** Department of Mechanical and Aerospace Engineering, University of Strathclyde, James Weir Building, 75 Montrose Street, Glasgow G1 1XJ, UK; balagopal.santhosh@strath.ac.uk

**Keywords:** particle dynamics, vibrational flow, inertial effects, numerical simulation, Eulerian–Lagrangian approach

## Abstract

Over the last decade, numerical simulations and experiments have confirmed the existence of a novel class of vibrationally excited solid-particle attractors in cubic cavities containing a fluid in non-isothermal conditions. The diversity of emerging particle structures, in both morphology and multiplicity, depends strongly on the uni- or multi-directional nature of the imposed temperature gradients. The present study seeks to broaden this theoretical framework by further increasing the complexity of the thermal “information” coded along the external boundary of the fluid container. In particular, in place of the thermal inhomogeneities located in the center of otherwise uniformly cooled or heated walls, here, a cubic cavity with temperature boundary conditions satisfying the *D*_2_*h* (in Schoenflies notation) or “*mmm*” (in Hermann–Mauguin notation) symmetry is considered. This configuration, equivalent to a bipartite vertex coloring of a cube leading to a total of 24 thermally controlled planar surfaces, possesses three mutually perpendicular twofold rotation axes and inversion symmetry through the cube’s center. To reduce the problem complexity by suppressing potential asymmetries due to fluid-dynamic instabilities of inertial nature, the numerical analysis is carried out under the assumption of dilute particle suspension and one-way solid–liquid phase coupling. The results show that a kaleidoscope of new particle structures is enabled, whose main distinguishing mark is the essentially one-dimensional (filamentary) nature. These show up as physically disjoint or intertwined particle circuits in striking contrast to the single-curvature or double-curvature spatially extended accumulation surfaces reported in earlier investigations.

## 1. Introduction

The study of interactions between particles and fluids is extremely important in several fields, e.g., chemistry, medicine, pharmaceuticals, materials science and the energy sector just to cite the most important ones. In particular, in all these cases, typically a key area of focus is the ability to manage the separation, sorting, and (especially) self-organization of the particles being dispersed in (or carried by) a given fluid. Such interest stems from the important applications enabled accordingly. Just to cite a few examples, in the area of materials science these capabilities have significant implications for the design and development of different types of advanced metal alloys [1,2]. The control of particle behavior within fluid phases can be instrumental in refining the alloy microstructure, which directly impacts the mechanical, thermal, and electrical properties of the final product. Additionally, such precise control is crucial for the development of composite materials where the dispersion of particles in a matrix can enhance strength, conductivity, or corrosion resistance [3]. Similar concepts apply to emulsions [4] and other organic substances where a minority phase is dispersed inside a liquid permanently or for a certain time (as it is typically the case of protein crystals grown by the counter-diffusion technique [5]). Other applications can be found in medicine where, for instance, such abilities are crucial for isolating specific types of cells from the blood [6] or, vice versa, promoting their aggregation to obtain samples of living tissues [7].

It is also worth highlighting that, in the energy field, understanding and controlling these behaviors is crucial for optimizing processes in a wide range of emerging microscale technologies. For instance, in micro-scale energy conversion systems, particle-laden fluids are often subjected to coupled thermal and mechanical excitations, and their dynamic response can strongly influence the overall efficiency of energy conversion. The ability to predict and modulate particle migration, aggregation, or alignment under such conditions can lead to improved energy transfer mechanisms and more robust operational stability [8,9].

Similarly, in advanced microreactors and catalytic devices, precise control over particle distribution within the carrier fluid can dramatically affect reaction kinetics and selectivity. A uniform dispersion enhances surface accessibility and mass transport, whereas controlled aggregation (or local clustering) can be exploited to tailor reaction micro-environments or enhance cascade reactions [10,11,12].

Furthermore, in thermal management strategies for microscale energy devices, the transport of suspended particles can significantly influence both heat transfer efficiency and fluidic stability. The controlled motion and organization of particles under temperature gradients or flow perturbations can be leveraged to augment convective transport, delay instabilities, or even create adaptive thermal pathways within miniaturized systems. Altogether, the fine regulation of particle–fluid interactions at such scales represents a cornerstone for the next generation of energy, chemical, and biomedical technologies, where enhanced functionality often arises from the interplay between fluid dynamics, thermal transport, and particulate behavior [13].

Among the possible strategies inherent to exerting the sought control on the dispersed phase, one may cite methods relying on (1) Van der Waals forces, which, though weak, can guide the assembly of molecules and nanoparticles into precise configurations, often crucial for creating nanoscale materials with specific properties [14] or even become effective at larger scales in microgravity conditions [15]; (2) electric fields, by which complex arrangements such as colloidal crystals can be obtained [16]; (3) magnetic forces, which can cause magnetic particles to self-assemble into chains, rings, or other structures [17]; (4) optical trapping mechanisms, that is, using radiation pressure to push particles into “traps” or force them to take specific paths [18]. (5) capillary forces, driven by surface tension and related effects, which can guide liquid particles in liquid systems to migrate towards certain regions [19], (6) other similar non-equilibrium effects [20,21] and (7) phenomena relying on “active matter” [22], i.e., microorganisms, other biological entities, and artificial microscopic particles or robots, which are able to capture and utilize energy and information from their environment, and produce accordingly highly complex, finely coordinated forms of self-organization. Such methods typically display a varying degree of success depending on the specific application and the scale at which the controlling strategy has to be implemented. All of them, however, are affected by an important limitation, that is, the considered particles must allow the required non-equilibrium or energy-exchange effects at their interface or must be sufficiently sensitive to the intended steering principle, be it a magnetic, electric or optical (light) field.

Very recently, therefore, some effort has been put into the identification of more general principles which do not suffer from these limitations and may therefore be applied to any type of particle dispersion in a fluid. In particular, a very promising line of research is that dealing with the dynamics of “inertial particles” because these particles allow the existence of “attractors” or “attractee” inside the host liquid. More specifically, “inertial particle attractors” in liquid flow refer to specific locations where solid particles tend to gather over time. This phenomenon arises due to a fundamental difference between the nature of liquid motion and the behavior of transported dispersed solid matter itself. While a liquid medium can essentially be considered incompressible, as mathematically represented by the well-known condition of a zero divergence of velocity, this property does not extend to the dispersed solid phase. Inertial particles do not follow strictly the path of the carrier flow and accordingly the space among them can either diminish or increase in time. The slight deviations of their trajectories from those followed by the fluid accumulate over time, enabling the particle swarm to compress or expand. It is this deviation from the incompressibility constraint that ultimately enables the existence of the aforementioned attractors.

Notably, although the physical structures emerging in the three-dimensional (3D) physical space in correspondence of the attractors obviously exhibit a sensitivity to the particle mass and size, the properties of the underlying attractors are predominantly dictated by the existence of certain “repetitive” spatial and/or temporal behaviors in the carrier flow. This category of phenomena has been theoretically contextualized accordingly in a series of studies by various authors (see, e.g., [23,24,25,26,27,28]). This robust theoretical foundation has spawned various research avenues, each exploring different aspects of the abovementioned “repetitive” effects crucial for particle clustering. These behaviors typically manifest as enclosed “tubes” in space, acting as templates for particle accumulation, or cyclic phenomena in time.

Such concepts have been successfully applied to liquid bridges exhibiting time-periodic (unstable) Marangoni flow [29,30,31,32,33,34,35,36,37,38], as well as to cases where the cyclic nature of the fluid flow is artificially induced by imposing vibrations on a non-isothermal flow in the absence of gravity [39,40,41,42,43].

The effective existence of particle attractors in the latter case has been recently confirmed through the execution of dedicated experiments on board the International Space Station [44]. It is noteworthy that these phenomena are also contingent upon the spatial finiteness of the system. In other words, the interaction between particles and the boundary plays a crucial role in facilitating the clustering process too, and this is true for both thermocapillary and thermovibrationally induced particle structures, which confirms the validity of other theoretical principles often also invoked to explain self-organization [45].

We wish to highlight that a clear mark distinguishing these lines of inquiry from other particle aggregation dynamics in turbulent flow [46] resides in the high reproducibility and regularity of the emerging structures, which has even recently inspired attempts to apply principles from topological algebra and geometry to develop a broad classification for the collective structures, akin to what is done with “quadrics” in projective geometry [47] (as opposed to the random shape and position of inertial particle clusters in chaotic flows).

In this context, the current study can be seen as another example of research focusing on the use of externally applied vibrations to drive the phenomena of interest [43,44]. What makes this problem particularly unique, compared to other cases like the aforementioned liquid-bridge problem, is the added flexibility provided by the various degrees of freedom associated with the effectively used thermal boundary conditions. Although these phenomena were initially observed in a differentially heated cavity with one uniformly heated wall and one uniformly cooled wall [39,40], subsequent studies demonstrated that this configuration can yield a variety of particle structure shapes depending on the relative direction of the imposed temperature gradient and vibrations [41]. As there is no specific reason to limit the consideration to uniformly heated or cooled walls, this realization prompted some researchers [48,49,50] to investigate scenarios where thermal inhomogeneities were intentionally introduced on otherwise uniformly cooled or heated walls. In the present work, such a line of inquiry is further pursued through the consideration of cavities where non-isothermal conditions are obtained through differential heating of the various cavity corners. Given the complexity of the problem, the simulations are conducted in the framework of one-way coupled approach (corresponding to the “dilute dispersion” assumption) in order to filter out the symmetry-breaking instabilities of inertial nature originally identified by [51], and allow meaningful identification of the overarching particle attractors existing in the phase space.

## 2. Mathematical Model

### 2.1. Considered Geometry and Related Thermal Boundary Conditions

**Figure 1:**
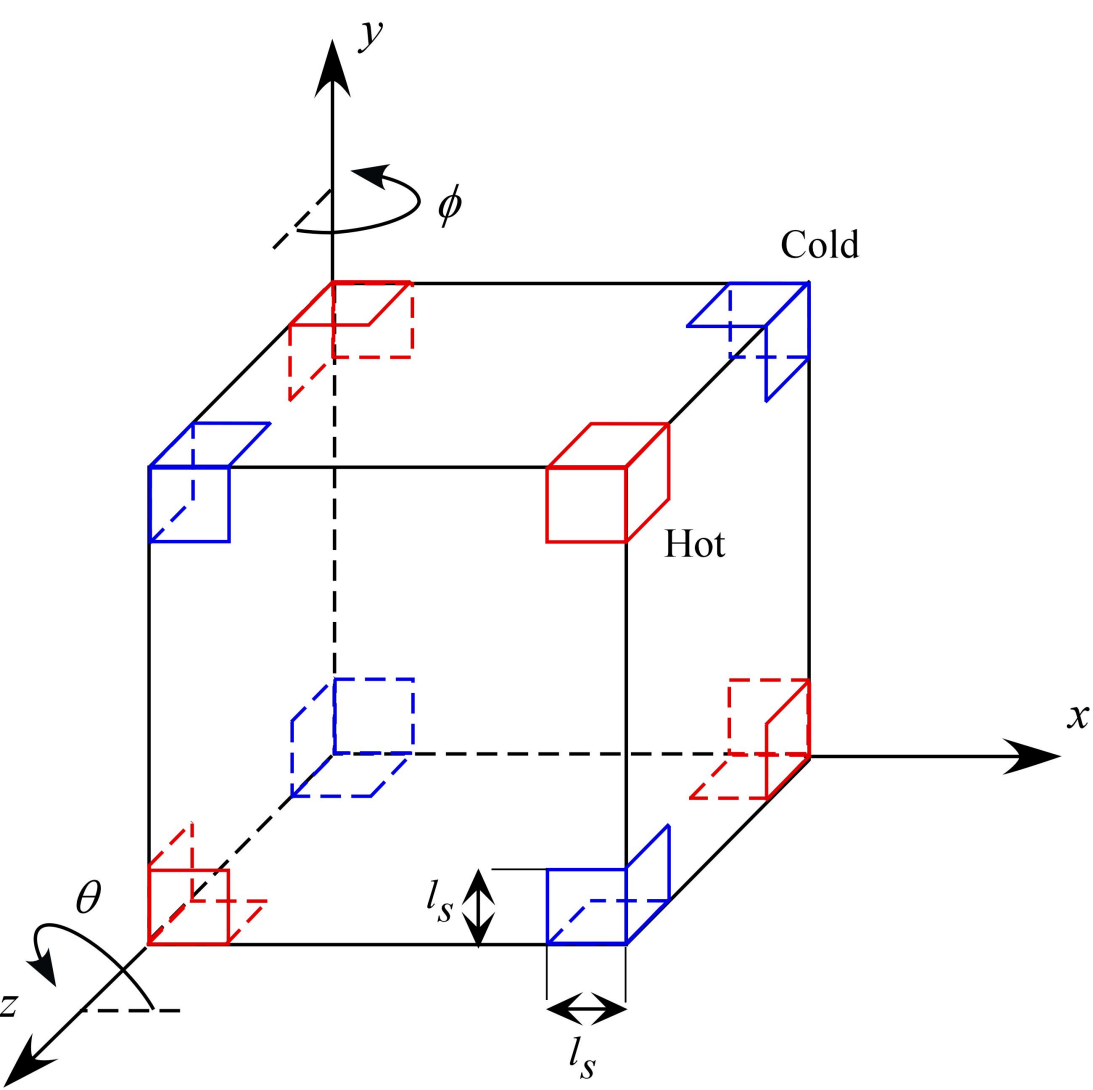
Sketch of the considered thermal boundary conditions (*D_2_h* configuration): each corner is heated (cooled) at the same temperature of the diagonally opposed corner pertaining to the same cube face (however, the corner temperatures are globally defined, and each face simply inherits that diagonal arrangement from the vertex coloring of the cube). The length *l*_s_ denotes the linear extension (along the cubic surface) of the thermally controlled area, *φ* is the angle formed by the projection of vibrations in the *xz* plane with the *z* axis, *θ* is the angle formed by the projection of vibrations in the *xy* plane with the *x* axis.

While we maintain the same cubic cavity examined in many prior studies, we largely increase the degree of thermal inhomogeneity of the fluid by considering sinks and sources of heat along the external boundary of the container specifically located at the cavity corners. Unlike all earlier configurations [39,40,41,42,49,50], therefore, the investigated system has no completely adiabatic walls. Moreover, the number of thermally controlled surfaces is expanded from the two walls originally considered by [39,40,41,42] or the four/eight boundaries of [49,50] to 24 distinct planar areas (refer to Figure 1). In such conditions, each wall features gradients of temperature directed simultaneously along different directions.

Just like the experiments recently conducted on board the International Space Station where a simple differentially heated cavity was considered [44,52], we assume the absence of gravity, positing that the phenomena occur under microgravity conditions.

The problem is tackled by examining the fluid and the movement of particles using established theoretical models [39,40,41,42,43,53]. Specifically, the Navier–Stokes equations are employed for the fluid phase, while the evolution of the dispersed solid phase is tracked through solution of the Maxey–Riley equation for each particle.

### 2.2. Driving Force and Governing Equations for the Fluid Phase

The behavior of the liquid is described accordingly through fundamental conservation laws for mass, momentum, and energy. Under the Boussinesq approximation, these equations simplify to:

(1) $$\underline{\nabla }\cdot \underline{V}=0$$

(2) $$\frac{\partial \underline{V}}{\partial t}=-\underline{\nabla }p-\underline{\nabla }\cdot \left[\underline{V}\underline{V}\right]+\mathrm{Pr}\nabla^{2}\underline{V}-\mathrm{Pr}Ra_{\omega }T\sin(\Omega t)\underline{\hat{n}}$$

(3) $$\frac{\partial T}{\partial t}+\underline{\nabla }\cdot \left[\underline{V}T\right]=\nabla^{2}T$$

In these expressions, *V*, *p*, and *T* represent the dimensionless velocity (with *u*, *v* and *w* as components along the *x*, *y* and *z* axes, respectively), pressure, and temperature. These equations closely resemble those traditionally used for buoyant flows caused by gravity on Earth. The only distinction is that here, the constant gravitational force is replaced by the time-dependent acceleration caused by the vibration applied to the fluid. This acceleration is modeled here as *bω*^2^sin(*ωt*), where *b* is the amplitude of vibration in meters and (*ω* = 2π*f*) (with *f* as the frequency in hertz) is the angular frequency of vibration [54,55]. The unit vector $\underline{\overset{⌢}{n}}$ simply defines the direction of vibration. The non-dimensional form of such equations stems from the assumption of canonical reference quantities, namely, the characteristic length *L*, the velocity scale *α*/*L*, the time scale *L*^2^/*α*, the pressure scale *ρα^2^*/*L^2^*, and the temperature difference Δ*T* = *T_hot_* − *T_cold_*. Related key dimensionless numbers include the Prandtl number:

(4) $$\mathrm{Pr}=\frac{\nu }{\alpha }$$

where *ν* represents the fluid’s kinematic viscosity, defined as the ratio of dynamic viscosity to density (*ν* = *μ*/*ρ*)), and *α* is the thermal diffusivity of the fluid. Moreover, the vibrational Rayleigh number *Ra_ω_* (similar to the classical Rayleigh number) is expressed as:

(5) $$Ra_{\omega }=\frac{\left(b\omega^{2}\beta_{T}\Delta TL^{3}\right)}{\nu \alpha }$$

where *β_T_* is the coefficient of thermal expansion. Additionally, the dimensionless angular frequency *Ω* of the vibrations is given by:

(6) $$\Omega =\frac{\omega L^{2}}{\alpha }=\frac{2\pi }{\tau }$$

where *τ* is the dimensionless vibration period.

Notably, the three above non-dimensional numbers can be combined into another useful non-dimensional group, generally known as the Gershuni number:

(7) $$Gs=\frac{\mathrm{Pr}}{2}{\left(\frac{Ra_{\omega }}{\Omega }\right)}^{2}$$

generally used to account for the strength of time-averaged effects in thermovibrational flow, i.e., the steady response that the fluid can provide to the application of an oscillatory force due to the intrinsically non-linear nature of the governing equations [39].

### 2.3. The Minority Phase

When the concentration of particles is relatively low, general consensus exists [56] that their influence on the fluid flow can be neglected, a scenario referred to as the “dilute distribution assumption”. Under this assumption, the problem can be analyzed using a one-way coupling approach, where particles are influenced by the fluid flow, but the fluid is assumed to remain unaffected by the particles. This approach has two primary advantages: it reduces computational costs and allows for the simulation of a large number of particles, making it easier to visualize the attractors. In this framework, the Lagrangian equation that governs the motion of individual particles can be cast in compact form as:

(8) dV¯pdt=1ξ+1/2−PrStf(Rep)V¯p−V¯+32dV¯dt+32V¯⋅∇¯V¯+ξ−1ξ+1/2γsin(Ωt)n^¯

where *γ* is a dimensionless parameter that accounts for the magnitude of the acceleration induced by the vibration:

(9) $$\gamma =\frac{b\omega^{2}L^{3}}{\alpha^{2}}$$

Moreover, in this context, *V_p_* denotes the velocity of the particle, while *f*(Re*_p_*) is a correction factor that accounts for the influence of the particle Reynolds number [57] on the drag force:

(10) fRep=1+0.15Rep0.687

The instantaneous Reynolds number for the particle is defined as:

(11) Rep=2RpρV¯−V¯pμ

To close the problem from a mathematical point of view, two particle-specific parameters must also be defined:

ξ = ρ_P_/ρ (12)

(13) $$St=\frac{2}{9}\frac{R_{P}^{2}}{L^{2}}$$

where *R_p_* is the particle radius, *ξ* is the ratio of particle density to fluid density, and *St* is the Stokes number.

In terms of model completeness and well-posedness, we wish also to highlight that no separate Lagrangian equation is used to describe heat transfer between the particles and the liquid because, in line with the approach of [51] and related justifications, we assume that given the small size of the considered particles, the solid phase remains in thermal equilibrium with the surrounding fluid.

### 2.4. Boundary Conditions

The boundary conditions for the considered system (*D_2_h* symmetry) can be sketched as follows:

(14a) *For ζ*_1_ = 0, 0 ≤ *ζ*_2_ ≤ *l_s_ and* 0 ≤ *ζ*_3_ ≤ *l_s_* ⟶ *T* = 0, *t* ≥ 0,

(14b) *For ζ*_1_ = 0, 1 − *l_s_* ≤ *ζ*_2_ ≤ 1 *and* 0 ≤ *ζ*_3_ ≤ *l_s_* ⟶ *T* = 1, *t* ≥ 0,

(14c) *For ζ*_1_ = 0, 0 ≤ *ζ*_2_ ≤ *l_s_ and* 1 − *l_s_* ≤ *ζ*_3_ ≤ 1 ⟶ *T* = 1, *t* ≥ 0,

(14d) *For ζ*_1_ = 0, 1 − *l_s_* ≤ *ζ*_2_ ≤ 1 *and* 1 − *l_s_* ≤ *ζ*_3_ ≤ 1 ⟶ *T* = 0, *t* ≥ 0,

(15a) *For ζ*_1_ = 1, 0 ≤ *ζ*_2_ ≤ *l_s_ and* 0 ≤ *ζ*_3_ ≤ *l_s_* ⟶ *T* = 1, *t* ≥ 0,

(15b) *For ζ*_1_ = 1, 1 − *l_s_* ≤ *ζ*_2_ ≤ 1 *and* 0 ≤ *ζ*_3_ ≤ *l_s_* ⟶ *T* = 0, *t* ≥ 0,

(15c) *For ζ*_1_ = 1, 0 ≤ *ζ*_2_ ≤ *l_s_ and* 1 − *l_s_* ≤ *ζ*_3_ ≤ 1 ⟶ *T* = 0, *t* ≥ 0,

(15d) *For ζ*_1_ = 1, 1 − *l_s_* ≤ *ζ*_2_ ≤ 1 *and* 1 − *l_s_* ≤ *ζ*_3_ ≤ 1 ⟶ *T* = 1, *t* ≥ 0,

with (*ζ*_1_, *ζ*_2_ *ζ*_3_) corresponding to (*z*, *x*, *y*), (*x*, *z*, *y*) and (*y*, *x*, *z*) for the three sets of opposing walls delimiting the cavity along different directions

(16a) *For ζ*_1_ = 0, 0 ≤ *ζ*_2_ ≤ 1 *and*
*l_s_* ≤ *ζ*_3_ ≤ 1 − *l_s_* ⟶ *∂T*/*∂ζ*_1_ = 0, *t* ≥ 0,

(16b) *For ζ*_1_ = 0, *l_s_* ≤ *ζ*_2_ ≤ 1 − *l_s_* *and* 0 ≤ *ζ*_3_ ≤ 1 ⟶ *∂T*/*∂ζ*_1_ = 0, *t* ≥ 0,

(16c) *For ζ*_1_ = 1, 0 ≤ *ζ*_2_ ≤ 1 *and*
*l_s_* ≤ *ζ*_3_ ≤ 1 − *l_s_* ⟶ *∂T*/*∂ζ*_1_ = 0, *t* ≥ 0,

(16d) *For ζ*_1_ = 1, *l_s_* ≤ *ζ*_2_ ≤ 1 − *l_s_* *and* 0 ≤ *ζ*_3_ ≤ 1 ⟶ *∂T*/*∂ζ*_1_ = 0, *t* ≥ 0,

with (*ζ*_1_, *ζ*_2_ *ζ*_3_) corresponding to (*z*, *x*, *y*), (*x*, *y*, *z*) and (*y*, *x*, *z*) for the three sets of opposing walls delimiting the cavity along different directions

In addition, a slip condition is imposed for the particles that allows them to move tangentially along the confining solid boundaries under the action of the carrier flow. This constraint is formally introduced by prescribing the particle radius as the minimum admissible distance between the particle centroid and the wall, thereby preventing unphysical particle–wall overlap while maintaining consistency with the governing hydrodynamic boundary conditions [39,40,41,42,49,50].

## 3. Numerical Method

The used numerical approach heavily depends on the interaction between pressure and velocity, which is why it is often referred to as pressure-based (solver) in many commercial CFD (Computational Fluid Dynamics) software packages (also known as “splitting methods for the incompressible Navier–Stokes equations”, the reader being referred to [58] for a more detailed illustration of this category of methods and related variants). The related underlying principles are briefly presented in the following with a specific focus on algorithm (implementation) aspects. In this regard, we start from the relatively simple concept that, in practice, the problem is tackled and solved through a multi-stage process. Specifically, this time-stepping algorithm is designed to integrate an alternate set of equations (mathematically equivalent to the original mass and momentum balance equations, though numerically different, as noted by [59]) to produce a velocity field that satisfies both the incompressibility condition and the momentum equation:

**Stage 1:** Initially, the momentum equation is integrated in a simplified, unphysical form where the pressure gradient term is omitted. This allows the straightforward derivation of a new velocity field based on the velocity at a given time instant:

(17) $$\frac{\partial {\underline{V}}^{*}}{\partial t}=\left[-\underline{\nabla }\cdot \left[\underline{V}\underline{V}\right]+\mathrm{Pr}\nabla^{2}\underline{V}-\mathrm{Pr}Ra_{\omega }T\sin(\Omega t)\underline{\hat{n}}\right]$$

Here, the right-hand side terms represent convective transport, diffusive transport, and momentum production, respectively (while as mentioned before, the pressure term has been intentionally excluded).

**Stage 2:** The physical relevance of the velocity is then recovered by reintroducing the previously ignored pressure term, leading to a correction equation:

(18) *V* =  V^*^ − Δ*t* ∇*p*

Substituting this corrected velocity into the mass balance equation yields an additional equation, which, after some mathematical manipulation, can be written as:

(19) $$\nabla^{2}p=\frac{1}{\Delta t}\underline{\nabla }\cdot {\underline{V}}^{*}$$

and can be used accordingly to determine the otherwise unknown pressure field.

From a purely mathematical standpoint, Equations (17) and (19) are of parabolic and elliptic nature, respectively, and they are typically solved using the effective fluid velocity at the boundary for Equation (17) (i.e., Dirichlet conditions corresponding to no-slip walls) and homogeneous Neumann boundary conditions (∂*p*/∂*n* = 0, where *n* is the direction normal to the solid wall, see, e.g., [60]) for Equation (19).

**Stage 3:** Equation (18) completes the algorithm loop by providing a formula for calculating the final, physically consistent velocity once the intermediate velocity *V^∗^* and pressure *p* are determined.

The velocity obtained through this method is then used to calculate the contributions at the right-hand side of Equation (8) thereby allowing the determination of the (Lagrangian) particle velocity field too. As those terms need to be evaluated at the particle’s instantaneous position, in particular, linear interpolation schemes have been exploited here to reconstruct the velocity and its derivatives at this location from the known nodal values at nearby grid points.

As the simulations have been conducted using the ANSYS Fluent (2024 R2) computational platform, additional useful details about the effectively exploited implementations can be provided as follows. The flow field has been computed with the pressure-based SIMPLE (semi-implicit method for pressure-linked equations) algorithm, by which pressure-velocity coupling is enforced through a semi-implicit formulation. Moreover, in ANSYS Fluent, a collocated mesh arrangement is employed, such that all dependent variables are defined at the centers of the control volumes.

The governing conservation equations for mass, momentum, and energy have been discretized using second-order upwind schemes for all convective fluxes, while diffusive terms have been approximated exclusively by standard central difference schemes. Within the Fluent solver, these equations are treated implicitly, with the sole exception of the buoyancy term, which is advanced explicitly. Convergence has been enhanced by means of a classical algebraic multigrid technique, operated with standard settings and a Gauss–Seidel smoothing procedure.

Additional details are also needed for the particle motion equation. This equation has been integrated in its original form using a fifth order Runge–Kutta method. In Ansys Fluent, the particle velocity appearing in the drag force is handled implicitly, whereas all remaining force contributions are evaluated explicitly.

For all simulated cases, the time step has been selected so as to resolve each vibration period with no fewer than forty temporal increments. The related computational grids have been tailored to thermovibrational flow regimes following the indications originally provided by [39,40]. Put simply, under the present conditions, the emerging flow structures are relatively smooth and spatially extended rather than confined near walls. As a result, the mesh can be designed without the dense clustering typically required to resolve thin viscous or thermal boundary layers, while still capturing all relevant dynamical features with accuracy and computational efficiency (accordingly, grids with 64,000 elements have been employed).

We also wish to highlight that, for the first time with respect to earlier efforts [39,40,41,42,43,53], in this study we have tracked an important global Lagrangian parameter, namely, the so-called accumulation measure [61] defined as:

(20) $$K(t)=\frac{1}{2(N_{part}-\tilde{n})}\sum_{i=1}^{N_{cell}}\left|k_{i}-\tilde{n}\right|$$

where *N*_cell_ is the total number of computational cells, $\tilde{n}$ is the average number of particle in each cell (*N_part_*/*N*_cell_) and *k_i_* is the effective number of particles in the generic cell *i*. This parameter can thus be interpreted as the normalized sum of deviations in particle count, *k_i_*(*t*), across the entire computational domain, relative to the average particle number in each cell. In essence, it quantifies the degree of particle accumulation, where the limiting values of 0 and 1 represent two extreme cases: 0 corresponds to a perfectly uniform distribution of particles throughout the domain, while 1 indicates that all particles are concentrated within a single computational cell. In the following (Section 4) it is used to quantitatively substantiate the influence of various parameters on the structure formation time and related compactification (the higher *K*, the larger the degree of compactification displayed by the emerging structures). Finally, before starting to deal with the main findings of this study, we wish to recall once again that, as explained at the beginning of Section 2.3, a one-way coupled approach means that the dispersed mass is expected to have no influence on fluid flow. Therefore, the high number of particles visible in the numerical simulations presented in Section 4 is used solely to detail the particle attractors: the higher the number of particles, the better their ability to reveal the underlying “attractee”, their number however being irrelevant to the overall dynamics.

## 4. Results

In line with the experiments recently conducted on board the International Space Station [44], we consider a cubic container with size 1 cm, ethanol as the working liquid (Pr = 18) and particles with diameter *d ≅* 75–90 μm (average *St ≅* 3.78 × 10^−6^). The density of the dispersed solid phase is 1320 kg/m^3^ as opposed to the density of ethanol *ρ* = 789 kg/m^3^⟶*ξ* = 1.65. Similarly, space experiment representative values are used for the non-dimensional acceleration amplitude and the vibrational Rayleigh number (fixed to *γ* = 4.8 × 10^8^ and *Ra_ω_* = 2 × 10^5^, respectively), while the non-dimensional angular frequency is allowed to span the interval 1 × 10^3^ ≤ *Ω* ≤ 2 × 10^3^. As already outlined before, while in the space experiments the system was subjected to a mono-dimensional temperature gradients imposed by differentially heating two opposing walls, here, a much more complex thermal environment is obtained through the involved (thermal) information encoded on the external boundary (see again Figure 1). Additional variables controlling the system response is the linear extension of the thermally controlled areas (*l*_s_), varied in the range 0.1 ≤ *l*_s_ ≤ 0.5, and the direction of vibrations with respect to the *x*, *y* and *z* axes (uniquely defined by assigning proper values to the *θ* and *φ* angles shown in Figure 1).

### 4.1. Unidirectional Temperature Gradient

To facilitate a thorough comprehension of this intricate topic, we adopt a methodical, deductive approach, that is, we start from simplified situations in terms of thermal boundary conditions, and progressively introduces systems of increasing complexity. Each successive layer builds upon the preceding one, allowing the reader to gradually assimilate more sophisticated ideas while retaining a firm grasp of the underlying principles.

Along these lines, first we consider the canonical configuration with two differentially heated opposed walls, these being uniformly heated and cooled, respectively, and vibrations (directed along the *x* direction) perpendicular to the resulting uni-dimensional temperature gradient (the *y* direction).

**Figure 2:**
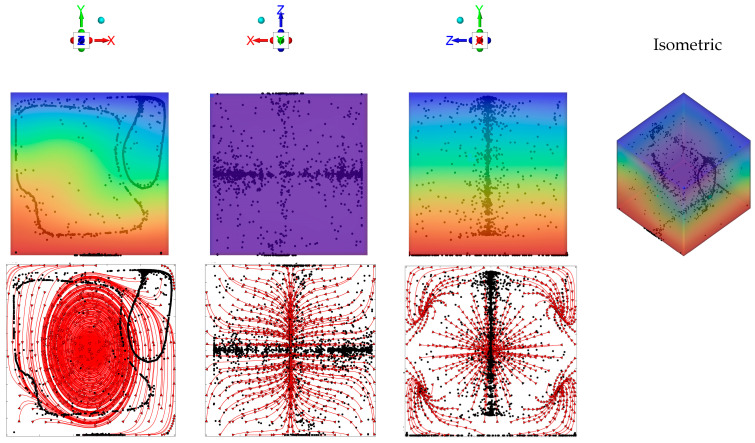
Snapshots of emerging particle patterns for the case of unidirectional temperature gradient produced by differentially heating two opposing walls while maintaining the other boundaries adiabatic (*Ω* = 1.5 × 10^3^, *Gs* = 1.6 × 10^5^, vibrations along the *x* axis, i.e., *φ* = 90°*, θ* = 0°). First row: temperature and particle distributions (perspectives perpendicular to the *xy*, *xz* and *yz* planes, respectively, and related isometric 3D view—Color legend: Red: Hot, Blue: Cold, Black: Solid matter). Second row: corresponding time-averaged fluid velocity field (red lines) in the same midplanes.

What stands immediately out from Figure 2 (first row, first panel) is that, although only the related “directrices” can be recognized, the emerging structures are essentially two cylinders [39,47]. From a purely geometric standpoint, a cylinder can be generated by translating a closed curve, called the directrix, along a fixed straight line, known as the generatrix or axis direction (as the curve moves parallel to this direction without rotation or deformation, every point on the curve traces a line segment parallel to the axis, forming a continuous surface). In our case, the directrix curves are located in the plane containing the temperature gradient and vibrations (the *xy* plane), while the axis of the structures is perpendicular to such a plane (*z* axis).

Notably, the considered case falls into the same regime originally identified by [42], i.e., a situation where, given the relative high value of the Gershuni number (*Gs* ≥ O(10^4^)), a significant (steady) time-averaged flow is produced in addition to the instantaneous one (second row of Figure 2). This steady flow is responsible for the continuous transfer of particles from one structure to the other until a state is attained where the two particle accumulations display an evident asymmetry, i.e., there is a significant variation in the extension of the related directrix (Figure 2, first column) [42].

Remarkably, the same (convective) effect is also responsible for a compression of the structures along their axis, i.e., along the generatrix (see Figures 10 and 11 in [42]). The same tendency was also scrutinized in the study by [62] (see Figures 5–7 in their work). In the present case, it is very evident in the second column of Figure 2 (i.e., the *xz* plane), where all the streamlines of the time-averaged flow clearly tend to transport all particles toward the *xy* midplane.

**Figure 3:**
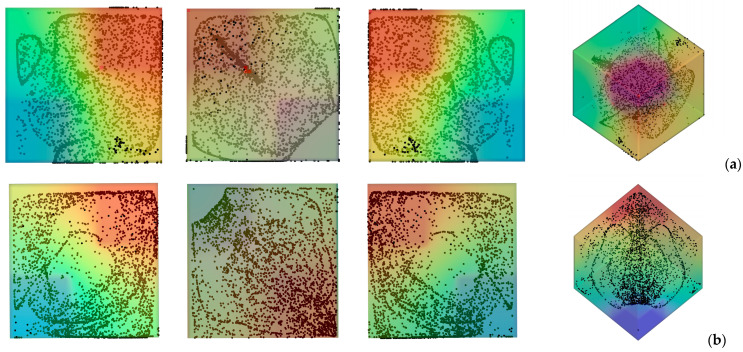
Snapshots of temperature distribution and related particle patterns (perspectives perpendicular to the *xy*, *xz* and *yz* planes, respectively, and related 3D view—Color legend: Red: Hot, Blue: Cold, Black: Solid matter) for the case of differentially heated diagonally opposed corners (*Ω* = 1.5 × 10^3^, *Gs* = 1.6 × 10^5^, *ls* = 0.4) (**a**) vibrations perpendicular to the *yz* plane (*φ* = 90°*, θ* = 0°); (**b**) vibrations perpendicular to the temperature gradient (*φ =* 45°, *θ* = 45°).

Figure 3 deals with the slightly more complex situation in which, even though the system is still subjected to a uni-direction temperature gradient; however, this is obtained by differentially heating two opposing corners of the cavity while keeping all the remaining boundaries adiabatic. In particular two situations are considered, namely, the case with vibrations still directed along the *x* axis (*φ* = 90°*, θ* = 0° for which vibrations are no longer perpendicular to the imposed temperature gradient, Figure 3 first row) and the configuration with vibrations rotated in such a way to make them again perpendicular to ∇*T* (*φ =* 45°,*θ* = 45°, Figure 3 second row).

**Figure 4:**
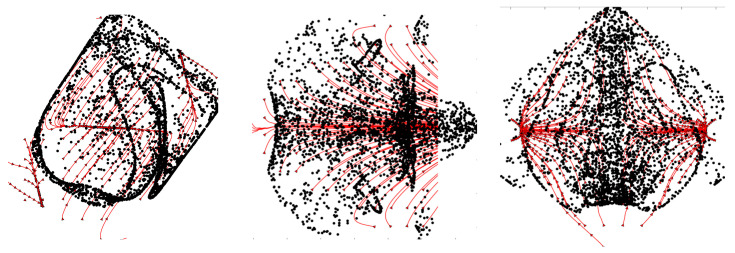
Particle distribution (perspectives perpendicular to the *xy*, *xz* and *yz* planes, respectively) and related time-averaged flow for the same conditions of Figure 3b.

**Figure 5:**
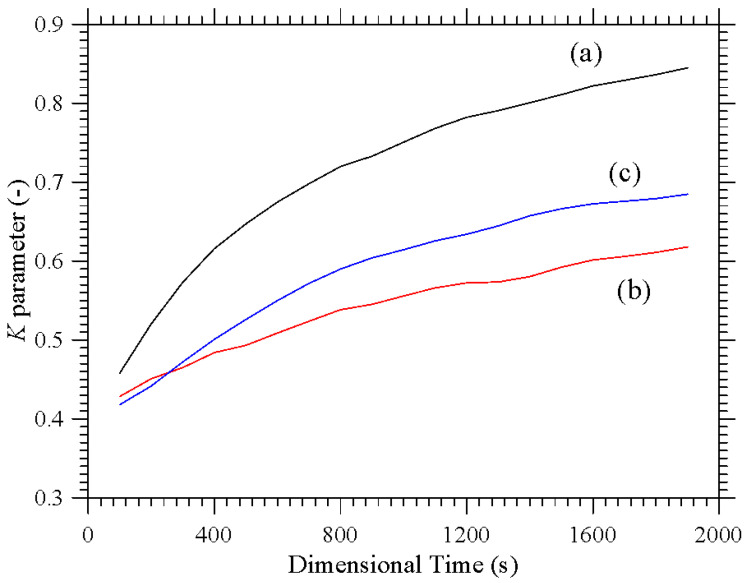
*K* parameter as a function of time for configurations with uni-directional temperature gradient: (**a**) Uniformly Heated/cooled opposed walls with vibrations perpendicular to the temperature gradient (conditions corresponding to Figure 2), (**b**) Differentially heated diagonally opposed corners with vibrations non-perpendicular to the temperature gradient (conditions corresponding to Figure 3a), (**c**) Differentially heated diagonally opposed corners with vibrations perpendicular to the temperature gradient (conditions corresponding to Figure 3b).

Taken together, these two cases are instructive, as they reveal the significant increase in complexity induced by a change as apparently innocuous as using corners instead of planar surfaces to thermally control the flow within cavity. Interestingly, while in the first case, the emergence of compactified closed particle circuits can still be identified in the *xy* and *xz* planes, in the second situation, the distribution of particles is more complex, which we ascribe to the very complex configuration taken by the time-averaged flow in this case (Figure 4). In this case well-defined particle circuits can be clearly recognized only if the 3D view is considered (Last panel of Figure 3b). The impact on the degree of accumulation measured by the parameter *K* (Equation (20)) is not trivial too (Figure 5).

While moving from the configuration with uniformly heated/cooled opposing walls (Figure 2) to that with two differentially heated corners and vibrations still directed along the *x* axis (Figure 3a) has clearly a negative effect in terms of compactification (as revealed by the related decrease in *K*), making the vibrations again perpendicular to the prevailing temperature gradient (Figure 3b) can mitigate this trend (leading to intermediate values of the *K* parameter, corresponding to the branch (c) in Figure 5).

### 4.2. D_2_h Symmetry: Influence of Thermally Controlled Area Extension

**Figure 6:**
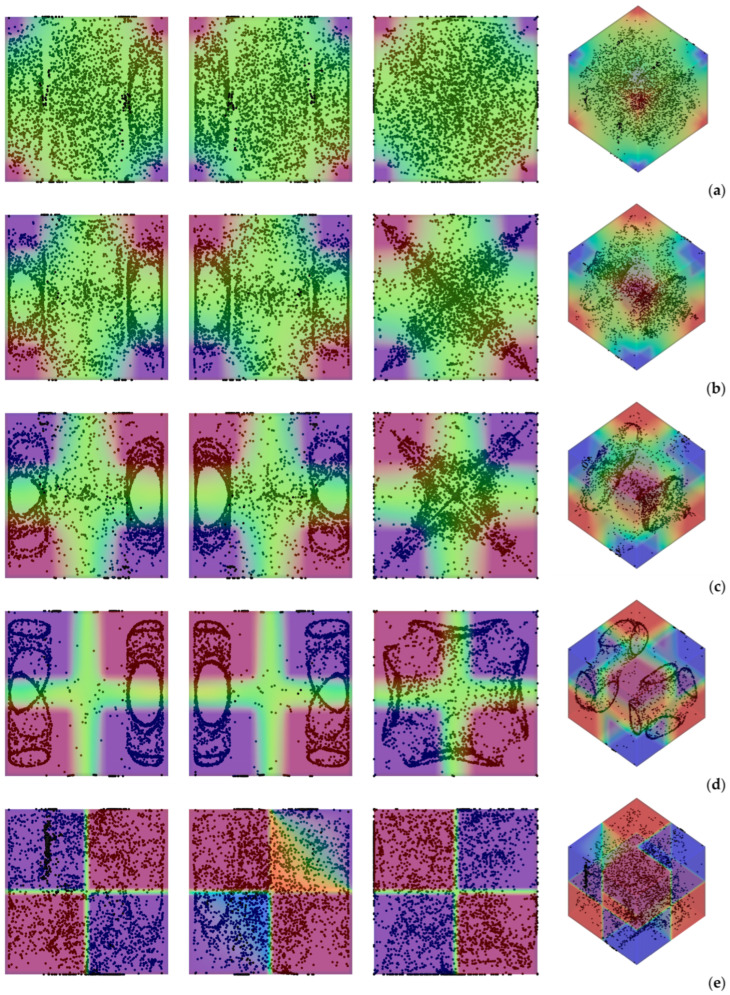
Snapshots of temperature distribution and related particle patterns (perspectives perpendicular to the *xy*, *xz* and *yz* planes, respectively, and related 3D view—Color legend: Red: Hot, Blue: Cold, Black: Solid matter) for *Ω* = 2 × 10^3^, *Gs* = 9 × 10^4^, *φ* = 90°, *θ* = 0° and different values of *l*_s_: (**a**) *l*_s_ = 0.1, (**b**) *l*_s_ = 0.2, (**c**) *l*_s_ = 0.3, (**d**) *l*_s_ = 0.4, (**e**) *l*_s_ = 0.5.

Having clarified the non-trivial effect that the thermal boundary conditions can have on the emerging dynamics even if the simplified case with uni-directional temperature gradient is considered, we now switch to the much more complex (multi-gradient) configuration with the *D_2_h* symmetry. In particular, first we explore the influence of the parameter *l*_s_, i.e., the linear extension of the thermally controlled areas in the “canonical” case with vibrations perfectly perpendicular to one of the walls. Along these lines, Figure 6 shows the outcome of the numerical simulations for the case *Ω* = 2 × 10^3^, and vibrations aligned again with the *x* axis (*φ* = 90°,*θ* = 0°).

As a first glimpse into this figure would immediately confirm, the most striking distinguishing feature of particle structures is still their relatively “filamentary” nature. Unlike quadrics or other closed surfaces observed in earlier studies where smaller values of the Gershuni number were considered (Gs ≅ O(10^3^)), in this case (Gs ≅ O(10^5^)), the particles accumulate along closed loops resembling (with the due differences) those produced by inertial particle dynamics in liquid bridges under the effect of Marangoni flow [29,30,31,32,33,34,35,36,37,38].

**Figure 7:**
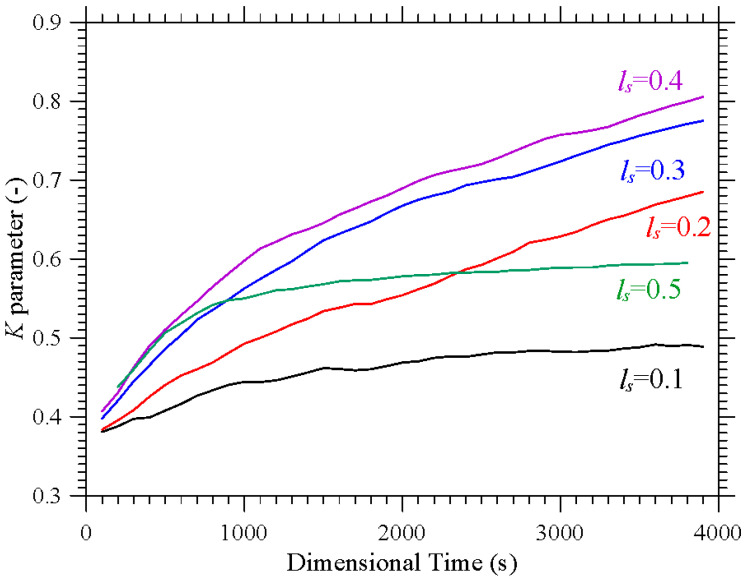
Evolution in time of the *K* parameter corresponding to the same conditions considered in Figure 6.

In particular, as also made evident by the complementary information reported in Figure 7, the compactification (i.e., the degree of accumulation) displayed by the formations depends significantly on the extension of the thermally controlled areas. While for *l*_s_ = 0.1 and *l*_s_ = 0.5, the emerging structures are hardly recognizable (*K* ≅ 0.48 and *K* ≅ 0.6 at *t* ≅ 4000 s, respectively), an interval of *l*_s_ exists where their topology can be clearly discerned (0.2 ≤ *l*_s_ ≤ 0.4 ⟶ 0.7 ≤ *K* ≤ 0.8). Specifically, in this range, several distinct, elliptical, or ring-like loops of particles can be recognized. As an example, Figure 6d (*l*_s_ = 0.4) shows eight independent loops, organized in two groups of four, located at the opposite ends of the cubic cavity. Each loop appears as an independent, closed structure, with no visible intersections among them. Moreover, each loop or particle circuit is approximately planar with its plane being parallel to the closest solid wall. However, for a smaller *l*_s_ (e.g., *l*_s_ = 0.3 in Figure 6c), the loops apparently coalesce into a cross-shaped accumulation structure, which becomes even thinner for *l*_s_ = 0.2 (Figure 6b).

Additional insights into these phenomena can be directly gathered from the aforementioned Figure 7 where they are characterized from a purely temporal standpoint through the related *K* parameter. This figure further confirms that accumulation is maximized for *l*_s_ = 0.4, while, consistent with the barely visible formations in Figure 6a, the configuration with *l*_s_ = 0.1 represents the worst case (*K* ≅ 0.48 after 4000 s, compared with *K* ≅ 0.81 for *l*_s_ = 0.4). The major significance of this figure, however, lies in its ability to show that although the transition from a cavity with two uniformly heated and cooled walls to one in which the temperature gradient is imposed between two diagonally opposite corners can cause a reduction in *K* (see again Figure 5), values of *K* even higher than 0.8 can be recovered if a multi-gradient configuration is adopted (upper branch in Figure 7).

### 4.3. Frequency Effects

**Figure 8:**
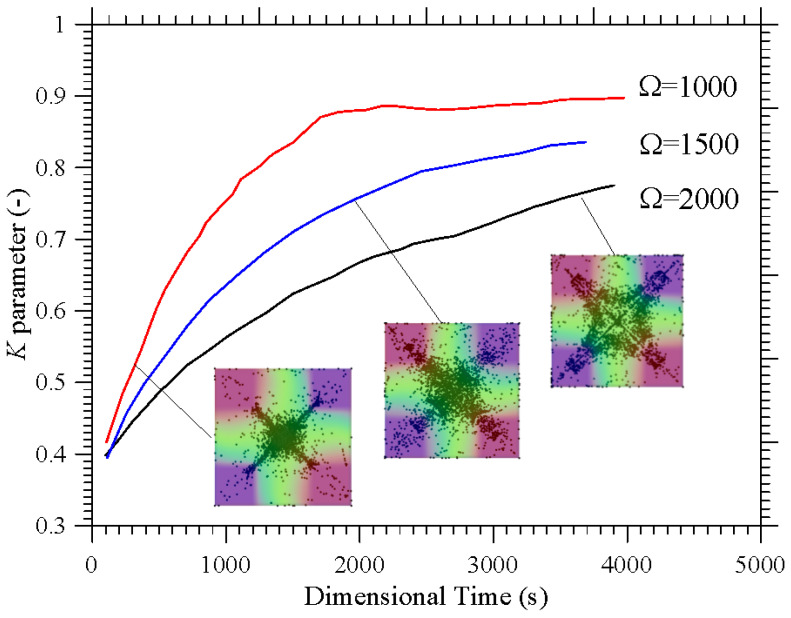
Temporal evolution of the *K* parameter for *l*_s_ = 0.3 and different values of the non-dimensional angular frequency *Ω.*

The frequency of the imposed vibrations can also have an impact on these mechanisms. This is shown in Figure 8 in terms of the *K* parameter for a representative (intermediate) value of *l*_s_ (*l*_s_ = 0.3).

**Figure 9:**
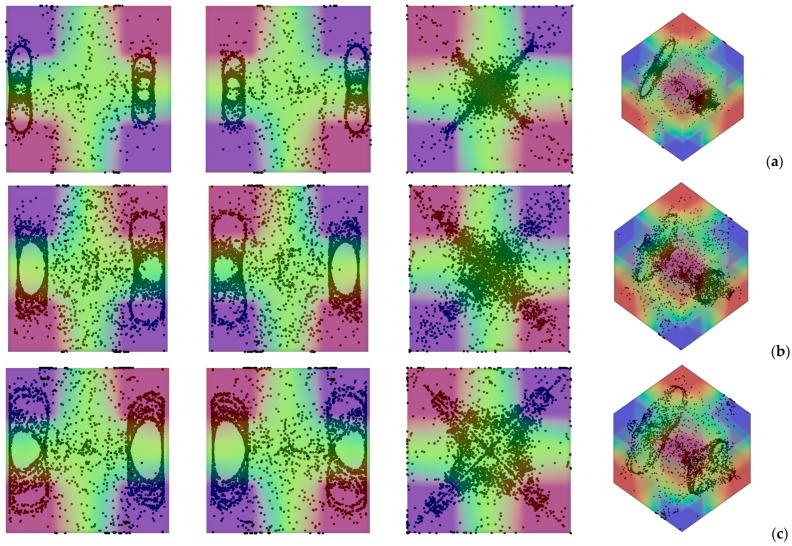
Snapshots of temperature distribution and related particle patterns (perspectives perpendicular to the *xy*, *xz* and *yz* planes, respectively, and related 3D view—Color legend: Red: Hot, Blue: Cold, Black: Solid matter) for *l*_s_ = 0.3, *φ* = 90°, *θ* = 0° and different values of *Ω*: (**a**) *Ω* = 1 × 10^3^, *Gs* = 3.6 × 10^5^, (**b**) *Ω* = 1.5 × 10^3^, *Gs* = 1.6 × 10^5^, (**c**) *Ω* = 2 × 10^3^, *Gs* = 9 × 10^4^.

As quantitatively substantiated in this figure, a decrease in *Ω* can significantly speedup the particle accumulation process, as witnessed by the relative position of the different curves (the branch for *Ω* = 2000 lying entirely below that for *Ω* = 1000). The related impact on formation size can be appreciated in Figure 9 where it can be seen that an increase in *Ω* (a decrease in *Gs* for a fixed value of the vibrational Rayleigh number *Ra_ω_* = 2 × 10^5^) essentially leads to more spatially extended structures. While on the one hand these findings support correlations identified in earlier studies (e.g., [42,47]) where the structure formation time was found to scale with the square of the non-dimensional angular frequency, on the other hand they add a new dimension to the problem by showing that the characteristic structure size (found to be almost independent from *Ω* and *Ra_ω_* in those works), can become dependent on the ratio between these two parameters (as formalized through Equation (7)) if the Gershuni number takes relatively high values. A simple rationale for this behavior can be identified in the compressive action that, as illustrated in Section 4.1 for simpler thermal boundary conditions, the time-averaged flow can exert on the emerging structures.

### 4.4. Varying the Direction of Vibrations

This section is dedicated to the effect of another important influential factor, that is, the direction of vibrations (uniquely identified through the two angles *φ* and *θ* defined in Figure 1). Since past studies have revealed significant variations in the morphology of the emerging formations even with thermal boundary conditions as simple as uniformly heated wall and an opposing uniformly cooled wall [39,41,47], the reminder of this study is entirely dedicated to this specific aspect. In particular, we concentrate on a representative value of *Ω* (*Ω* = 1500) and the conditions for which more defined structures have been observed in the canonical situation with *φ* = 90°, *θ* = 0° examined in the two earlier sections, namely, 0.2 ≤ *l*s ≤ 0.4.

**Figure 10:**
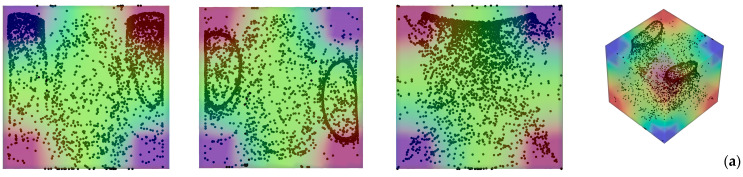

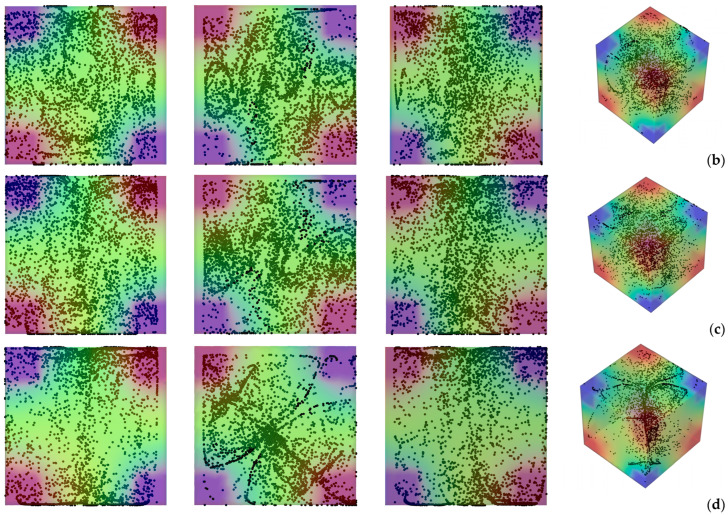
Snapshots of temperature distribution and related particle patterns (perspectives perpendicular to the *xy*, *xz* and *yz* planes, respectively, and related 3D view—Color legend: Red: Hot, Blue: Cold, Black: Solid matter) for *l*_s_ = 0.2, *Ω* = 1.5 × 10^3^, *Gs* = 1.6 × 10^5^, *θ* = 0° and different values of *φ*: (**a**) *φ* = 10°, (**b**) *φ* = 20°, (**c**) *φ* = 30°, (**d**) *φ* = 45°.

**Figure 11:**
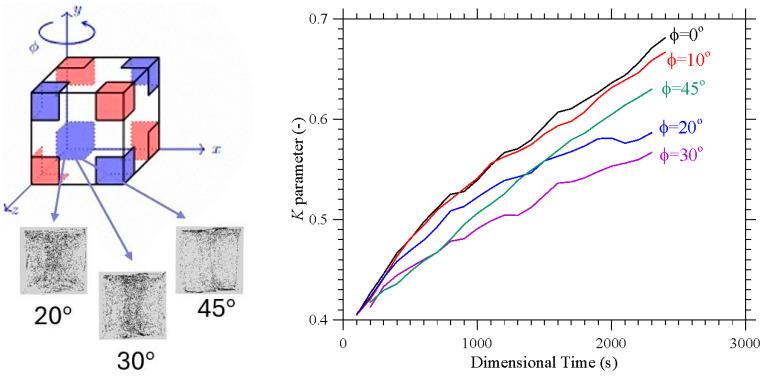
Temporal evolution of the *K* parameter for the same conditions considered in Figure 9 (*l*_s_ = 0.2, *Ω* = 1.5 × 10^3^, *Gs* = 1.6 × 10^5^, *θ* = 0°, 10° ≤ *φ*≤ 45°).

In such a context, Figure 10 reveals that even a minimal variation in the angle *φ*, while maintaining *θ* = 0°, can cause a dramatic variation in the position and morphology of the emerging particle formations. As an example, in place of the two cross-shaped thin structures seen in Figure 6b for *l*_s_ = 0.2, *θ* = 0° and *φ* = 90°, two compressed tubular structures with axis perpendicular to the *xz* plane are obtained for *θ* = 0° and *φ* = 10° (Figure 10a). For *φ* = 20° and *φ* = 30° the particle formation evolves into an irregular and very diffused central column (see plane *yz* in Figure 10b,c, respectively; the reader being also referred to Figure 11). The complementary information collected in Figure 11 confirms that these structures do not have high *K* parameter values, thereby reinforcing the interpretation that increasing the angle of inclination exerts a detrimental influence on the accumulation mechanism. In other words, as the inclination becomes more pronounced, the system’s capacity to sustain coherent particle clustering diminishes. This outcome stands in stark contrast to the trends reported in the two-dimensional numerical investigation with corner-heated thermal boundaries conducted in [48], where larger inclination angles were shown to enhance, rather than suppress, the organization and persistence of accumulative patterns. The discrepancy suggests that the transition from a two-dimensional to a three-dimensional configuration introduces additional geometric and dynamical complexities, such as asymmetric gravitational components, lateral confinement effects, and modified convective pathways that substantially alter the balance between particle accumulation and dispersion mechanisms within the flow.

A particularly intriguing and unexpected behavior emerges at an inclination angle of 45° (Figure 10d). The slender, filamentary particle structures emerging in this case represent a transitional morphology between dispersed and fully clustered states. Upon closer examination, they reveal a remarkable degree of spatial order, manifesting a quasi-symmetric arrangement that aligns preferentially with the direction of the imposed vibrations (the *x* axis in the *xy* plane).

**Figure 12:**
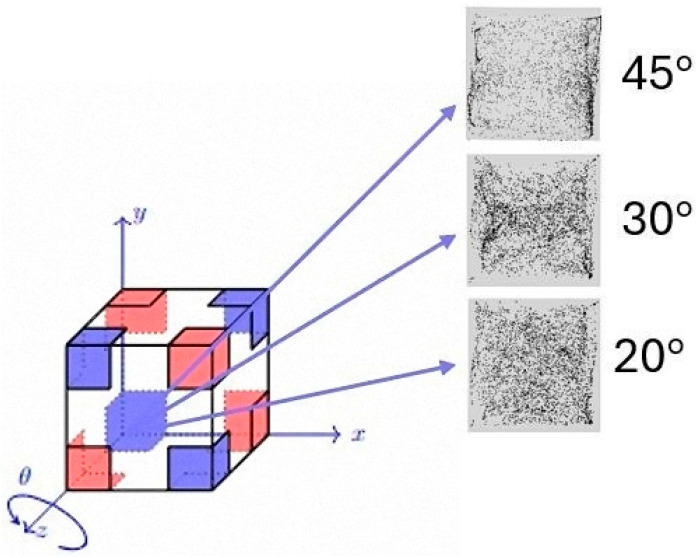
Temporal evolution of the *K* parameter for *l*_s_ = 0.2, *Ω* = 1.5 × 10^3^, *Gs* = 1.6 × 10^5^, *φ* = 90°, 10° ≤ *θ* ≤ 45°).

The next figure of the sequence, i.e., Figure 12 is used simply to show that, given the problem symmetries, equivalent variations in the angle *θ* while keeping *φ* fixed to 90°, produce analogous changes in the particle clustering behavior, the only difference being a 90° rotation of the entire pattern (compare Figure 12 with Figure 11).

Along these lines, it is worth recalling some of the typical properties of the *D_2_h* symmetry (Schoenflies notation), which the thermal boundary conditions employed here satisfy. As initially explained in Section 2, we have considered a cube in which each face has two diagonally opposite corners kept at one temperature and the other two at a different temperature. This leads to opposite vertices of the cube sharing the same temperature, i.e., an arrangement that is invariant under inversion symmetry through the cube’s center (*x*_0_, *y*_0_, *z*_0_). This property can be represented mathematically as

(*x* − *x*_0_, *y* − *y*_0_, *z* − *z*_0_) → (*x*_0_ − *x*, *y*_0_ − *y*, *z*_0_ − *z*) (21)

The considered problem is also invariant under any 180° rotation about an axis through the midpoints of opposite edges, since such a rotation interchanges equivalent hot/cold positions in pairs. Therefore, the *D_2_h* group, also denoted as *mmm* in Hermann–Mauguin notation, possesses three mutually perpendicular twofold rotation axes and inversion symmetry through the cube’s center. Each 180° rotation interchanges equivalent hot and cold corners, preserving the overall pattern, while reflections swap diagonally opposed temperature pairs. This symmetry contrasts with the full cubic group O_h_ (48 operations), representing a reduced but still highly ordered state (8 operations).

**Figure 13:**
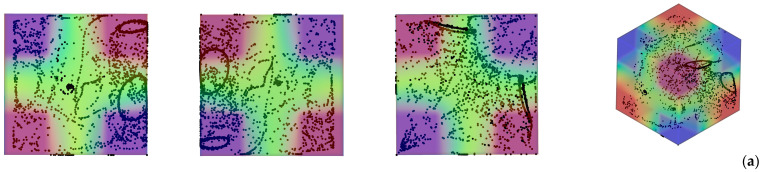

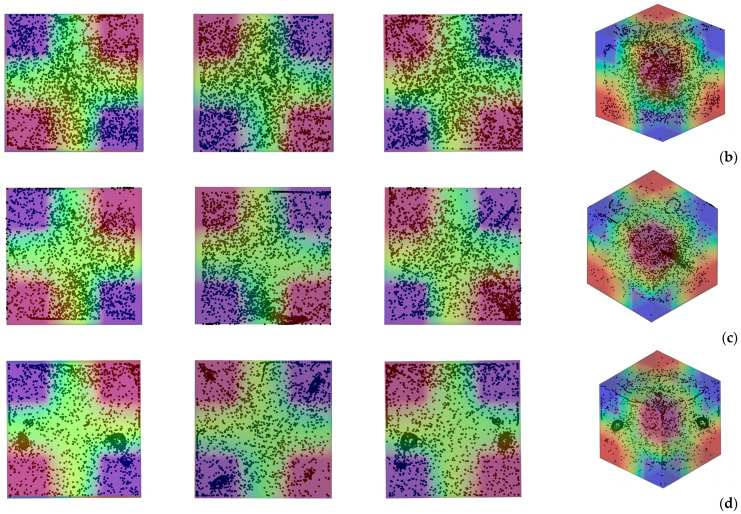
Snapshots of temperature distribution and related particle patterns (perspectives perpendicular to the *xy*, *xz* and *yz* planes, respectively, and related 3D view—Color legend: Red: Hot, Blue: Cold, Black: Solid matter) for *l*_s_ = 0.3, *Ω* = 1.5 × 10^3^, *Gs* = 1.6 × 10^5^, and different values of *φ = θ*: (**a**) *φ = θ *= 10°, (**b**) *φ = θ* = 20°, (**c**) *φ = θ* = 30°, (**d**) *φ = θ* = 45°.

**Figure 14:**
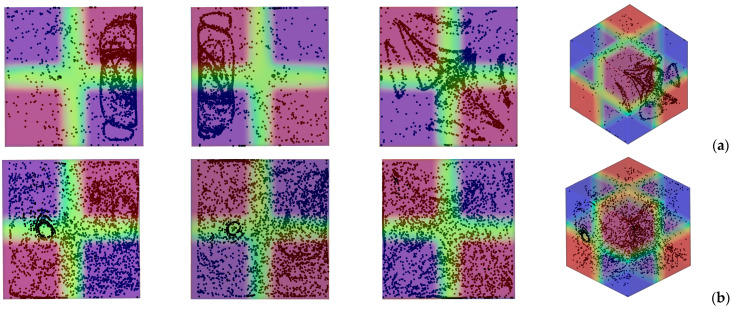

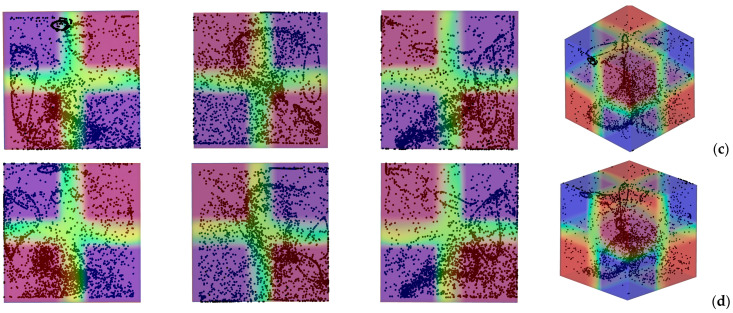
Snapshots of temperature distribution and related particle patterns (perspectives perpendicular to the *xy*, *xz* and *yz* planes, respectively, and related 3D view - Color legend: Red: Hot, Blue: Cold, Black: Solid matter) for *l*_s_ = 0.4, *Ω* = 1.5 × 10^3^, *Gs* = 1.6 × 10^5^, and different values of *φ = θ*: (**a**) *φ = θ* = 10°, (**b**) *φ = θ* = 20°, (**c**) *φ = θ* = 30°, (**d**) *φ = θ* = 45°.

To explore further the influence of this symmetry in terms of thermal boundary conditions on the emerging particle structures, in the following, other interesting variations are obtained by changing both *φ* and *θ*. In particular, for simplicity, here we limit ourselves to considering the situation in which the two angles assume the same value, i.e., *φ = θ* (Figure 13 and Figure 14 for *l*_s_ = 0.3 and 0.4, respectively).

Interestingly, for *l*_s_ = 0.3 and *φ = θ* = 10°, the two particle circuits previously seen in Figure 10a are still present. Their location, however, is completely different. In place of appearing near the top solid boundary, they are now located in proximity to (and intersect) a diagonal plane (Figure 13a). Although each circuit is almost planar, they are contained in two approximately perpendicular planes.

The columnar particle accumulation structures previously visible in Figure 10 (*yz* planes) for *φ =* 20°, and *φ =* 30° can no longer be recognized in the corresponding panels (b) and (c) of Figure 13. In these cases, a more or less diffused distribution of particles represents the typical outcome. However, an exception is once again represented by the 45° inclination (*φ = θ* = 45°) for which two dense particle circuits are formed (Figure 13d). Rather than being located in proximity to one of the planar solid boundaries, these occupy two opposite corners and are located midway within the cavity. Moreover, unlike the more spatially extended circuits found for the other cases, they display a relatively compact nature.

Notably, an increase in *l*_s_ can make the patterning behavior even more complex (see Figure 14). As an example, for *φ = θ* = 10° (Figure 14a), the two independent loops seen in Figure 13a are replaced by an involved multi-loop configuration. The particle patterns are oriented in multiple directions, with some following diagonal paths toward the corners.

Given the complexity of the system response in terms of particle patterning behavior and the seemingly endless variety of formations that can be obtained by changing the extension (*l*_s_) of thermally controlled planar surfaces and the direction of vibrations, we now refrain from expanding the catalogue of results and instead concentrate on interpreting the observed trends and developing a coherent rationale capable of tracking the evolution of particle structures from simple to progressively more complex configurations as the multiplicity of thermally controlled regions increases (Section 5).

## 5. Discussion

Clarifying the present dynamics and the underlying cause-and-effect relationships requires a brief excursus on earlier findings and related interpretations about this unique category of phenomena.

For an extensive description of the mechanism leading to particle structure formation, in particular, the reader may consider [39,47], where it was elucidated through a detailed examination of the evolving particle distribution in relation to the instantaneous velocity field generated by the combined influence of vibration and imposed thermal gradient.

In general, this phenomenon arises from a subtle synchronization between acceleration-induced inertial forces, which drive non-isodense particles to oscillate back and forth along the vibration axis, and the thermovibrational flow, whose convective and viscous effects periodically transport particles within the host fluid along different directions. The system boundaries (solid walls) are also integral to these dynamics as they provide spatial confinement and maintain, accordingly, the conditions necessary for the aforementioned synchronization to emerge. Equally essential is the temporally cyclic character of the thermovibrational flow, which enables the gradual accumulation of discrepancies between the trajectories of the solid particles and those of the surrounding fluid. Over successive cycles, these minute mismatches amplify, ultimately steering the dispersed phase toward a metastable configuration (represented mathematically by a non-solenoidal particle velocity field) wherein the particles collapse onto distinct surfaces or loci of accumulation. These regions act as sinks, i.e., closed spatial domains that allow particle entry but preclude their escape, thereby defining the final topology of the aggregated structure.

**Figure 15:**
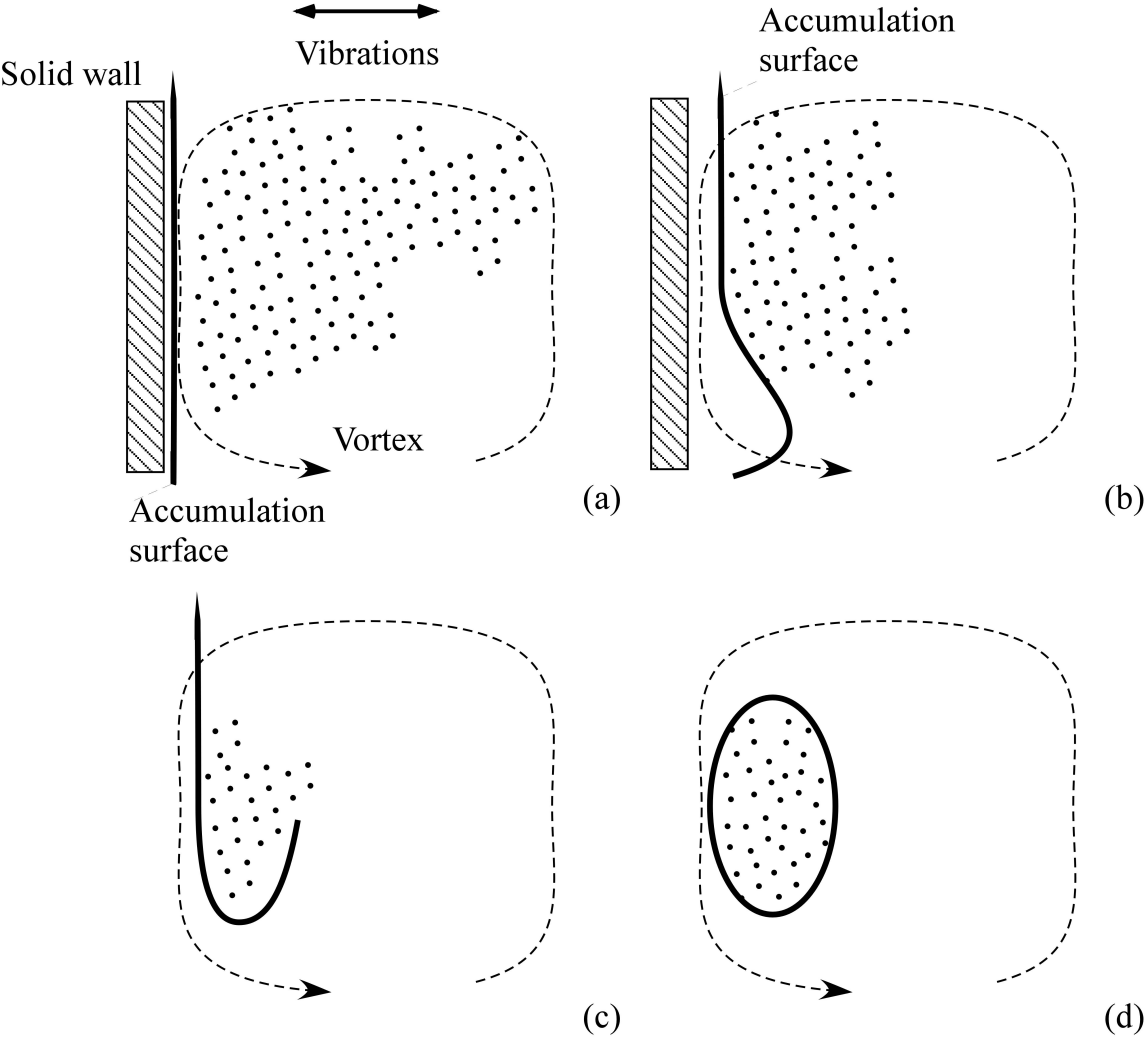
Schematic illustration of the process responsible for the formation of typical particle-dense boundary layers. The thick solid line represents regions of particle accumulation caused by repeated interactions between particles and the wall during vibration cycles. The dashed line (arrow) indicates the simultaneous thermovibrational flow, which develops alongside the clustering process: (**a**) straight vertical accumulation lines formed as a result of particles hitting the wall under the effect of vibration-induced body force, (**b**) accumulation line distorted due to its interaction with thermovibrational flow, (**c**) additional folding produced, (**d**) closed regions of particle accumulation emerge.

The decisive influence of confinement is particularly evident in the early stages of the formation process, as schematically shown in Figure 15. When a cluster of particles, subjected to a vibration-induced periodic force, approaches one of the system’s rigid boundaries, the particles tend to align and accumulate along the boundary’s orientation. Upon reversal of the periodic force, the particles are displaced in the opposite direction, leading to the emergence of narrow, particle-rich lines (in 2D) or surfaces (in 3D) that demarcate the interface between the particle-laden region and the adjacent particle-depleted (pure fluid) domain.

As the large-scale thermovibrational circulation develops, these dense lines or surfaces are advected by the instantaneous flow, stretching and bending into gracefully curved branches that delineate the evolving particle-rich zones. The ensuing patterning behavior is dictated by the progressive displacement of these concentrated surfaces or filaments away from the boundaries, accompanied by a folding process due to purely convective effects occurring in the fluid bulk (the thermovibrational flow itself).

Once established, these structures attain dynamical stability, representing the final, enduring patterns of accumulation. The subsequent temporal evolution of the system is characterized by gentle, periodic oscillations of the entire pattern, shifting alternately from left to right along the direction of vibrations in perfect synchrony with the external forcing [39,41].

When the thermal boundary conditions are kept simple, namely, when the system consists of a fluid-containing cubic cavity bounded by two opposite walls maintained at different but uniform temperatures such as that shown in Figure 2, the resulting thermovibrational flow field is generally characterized by a single dominant circulation cell. This roll periodically reverses its sense of rotation from clockwise to counterclockwise, and vice versa, in synchrony with the oscillatory forcing [39]. The flow pattern in this configuration is thus relatively simple and coherent: the entire liquid domain participates in a large-scale motion that alternately inverts direction as the imposed vibrational acceleration changes sign. Within such a regime, the coupling between thermal and vibrational effects yields a predictable oscillatory flow topology, which acts as the fundamental mode of the system.

When this idealized temperature distribution is perturbed by introducing localized spots of opposite thermal polarity at the centers of the two heated and cooled walls (a unimodal distribution of inhomogeneities such as that originally considered by [49]), the system shifts from a uni-directional gradient thermal configuration to a multi-directional one, and under the effect of the external forcing the internal flow reorganizes into a more intricate configuration. Under these modified boundary conditions, the thermovibrational velocity field within the plane defined by the direction of vibration and the imposed temperature difference typically divides into multiple recirculating structures, most often three parallel elliptic rolls of comparable strength, or a dominant central vortex accompanied by smaller, symmetrically positioned satellite rolls [49]. This transition from a single-cell to a multicellular structure implies a profound change in the underlying convective dynamics: the flow field becomes spatially fragmented, and the transport of suspended particles is modified accordingly.

In general, earlier efforts along these lines have shown that a clear inverse relationship emerges between the number of convective rolls and the characteristic scale of the resulting particle structures. Systems dominated by a single or few rolls tend to produce large, well-defined accumulation surfaces, while those exhibiting a higher number of rolls yield correspondingly smaller and more numerous structures. The conceptual ingredient needed to interpret this behavior is that the presence of multiple rolls divides the fluid domain into smaller convective compartments, consequently splitting the overall process of particle aggregation into different sub-regions. In contrast, a single-roll configuration preserves global coherence, enabling particles to cluster into fewer but larger accumulation regions that display remarkable geometric simplicity and temporal stability. Obviously, the morphology of these accumulation surfaces depends not only on the number of convective rolls but also on the geometric relationship between the imposed temperature gradients and the direction of vibrations. Accordingly, particles can organize locally along single-curvature surfaces like cylinders or cones, or double-curvature configurations (such as ellipsoids and hyperbolic paraboloids) [47] or even be compressed forming (degenerate) planes in the limiting condition where vibrations and the prevailing temperature gradient have the same direction [53].

Interestingly, Ref. [50] could show that a further increase in thermal complexity, achieved by considering a cubic cavity with two hot and two cold walls each perturbed at their centers by temperature inhomogeneities of opposite sign (a bimodal distribution relying on 8 distinct thermally controlled areas), leads to an even richer dynamical behavior. In this configuration, the number of rolls proliferates, producing a multicellular flow structure where vortices occupy eccentric positions. Instead of a single dominant convective axis, the system exhibits a network of interacting rolls whose centers shift away from the geometric center of the cavity. This eccentricity not only modifies the velocity field distribution but also introduces regions of intense shear and complex particle trapping mechanisms. In this conditions, the coherent accumulation surfaces characteristic of single- or double-curvature configurations gradually give way to filamentary structures [50]. In this regime, the particles no longer assemble into broad continuous surfaces but rather into elongated, thread-like formations that intertwine and interconnect throughout the volume.

Superimposed on these effects is the influence of time-averaged convection, which as shown by [42,62] for the cases of uniformly heated/cooled walls or walls with thermal perturbations of opposite sign at the center, respectively, generally tends to induce a compression of the otherwise spatially extended cylindrical structures along the direction of their generatrix.

In the light of the existing literature, we therefore ascribe the striking filamentary and intertwined nature of the observed particle patterns in the *D_2_h* configuration to the combined influence of the highly multicellular character of the instantaneous carrier flow and the presence of significant (non-negligible) time-averaged convection.

**Figure 16:**
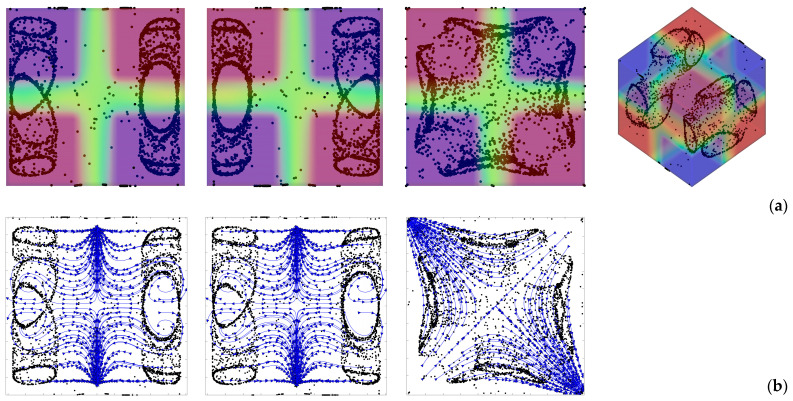

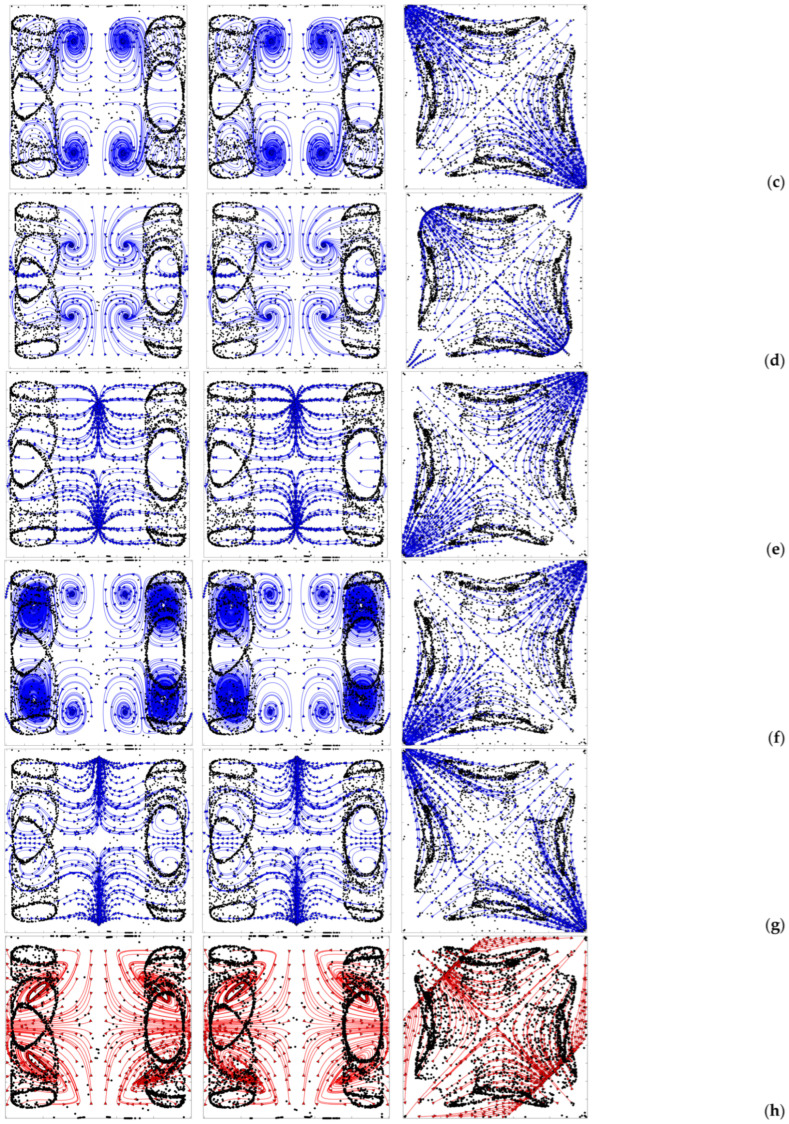
Particle patterns (perspectives perpendicular to the *xy*, *xz* and *yz* planes, respectively) for Case *Ω* = 2 × 10^3^, *Gs* = 9 × 10^4^, *φ* = 90°, *θ* = 0 and *l*_s_ = 0.4: (**a)** Corresponding temperature distribution, (**b**) instantaneous velocity field at *t* = *t_o_* + *τ*/6, (**c**) *t* = *t_o_* + 2*τ*/6, (**d**) *t* = *t_o_* + *τ*/2, (**e**) *t* = *t_o_* + 2*τ*/3, (**f**) *t* = *t_o_* + 5*τ*/6, (**g**) *t* = *t_o_* + *τ*, (**h**) Velocity field time averaged over *τ* (where *τ* = 2π/*Ω* is the period of vibrations).

In the following the validity of this interpretation is specifically demonstrated for a representative case with the *D_2_h* symmetry, namely, the case *Ω* = 2 × 10^3^, *Gs* = 9 × 10^4^, *φ* = 90°, *θ* = 0° and *l*_s_ = 0.4, as shown in Figure 16.

The alternating nature of the fluid motion supporting the particle accumulation mechanism depicted in Figure 15 can be clearly recognized in Figure 16b–g where the instantaneous flow is shown at 6 times evenly spaced within one vibration period. It can be seen that regions exist in proximity to the two lateral walls perpendicular to the direction of vibrations (the *x* axis in this case) hosting localized vortices (or curved streamlines) that periodically change their orientation (from clockwise to counter-clockwise or vice versa) and cause accordingly the formation of closed particle circuits in line with the arguments provided before. Remarkably such rolls are not visible only in *xy* planes; they also appear in *xz* planes, which explains why the particle structures are formed in both planes. Furthermore, the mirror symmetry of the instantaneous flow with respect to the central section in both *xy* and *xz* planes is the additional conceptual ingredient needed to understand why 4 particle circuits are formed at both ends (along the *x* direction) of the cavity.

Remarkably, the time-averaged flow shown in Figure 16h is also an important ingredient contributing to the overall dynamics. Indeed, it further contributes to cause the confinement of the emerging structures to the regions located in proximity to the two opposing walls perpendicular to vibrations as follows: on average, particle initially uniformly dispersed in the cavity tend to be transported along the positive or negative *x* direction from the central zone towards the two end regions of the cavity where they are trapped into circuits through the aforementioned mechanism involving particle accumulation along the wall and ensuing folding of the accumulation surface due to its interaction with the instantaneous flow.

Similar arguments could be developed for all the other cases treated in the present work (Figure 6, Figure 9, Figure 10, Figure 13 and Figure 14). To avoid duplications, however, we limit ourselves to highlighting that all these cases can be interpreted under the common umbrella depicted before, that is, the coexistence of numerous rolls, both instantaneous (continuously switching between clockwise and counterclockwise orientations over time) and time-averaged (steady), often with unequal strength and eccentric positions, which generate a complex lattice of local velocity gradients and shear layers. These act by repeatedly compressing, stretching and folding the particle accumulations surfaces initially caused by the particle–wall interaction under the effect of vibrations, promoting the formation of fine, intertwined filaments.

In an attempt to develop a unified discussion that also incorporates earlier results, and to articulate a rationale elucidating how particle structures transition from simple to increasingly intricate forms as additional thermally controlled segments are introduced along the system boundary, the following evolutionary progression can therefore be envisioned for the considered class of phenomena:

1. **Single Gradient Configuration**—One dominant convective roll; extended single-curvature or double-curvature accumulation surfaces; coherent global motion.
2. **Unimodal Perturbation**—Introduction of localized thermal spots; three to five rolls; fragmentation of the flow field; emergence of smaller surfaces with higher curvature.
3. **Bimodal Perturbation**—Two hot and two cold walls with opposite perturbations; numerous eccentric rolls; transformation of accumulation surfaces into partially disconnected patches; onset of filamentary traces.
4. ***D*****_2_*****h***** Symmetric Configuration**—Full thermal encoding on all walls; maximum roll multiplicity; complete fragmentation of continuous surfaces; stabilization of closed filamentary loops and intertwined structures.

Interestingly, this progression underscores a fundamental principle: increasing thermal complexity induces a dimensional reduction in the attractor morphology, driving the system from extended two-dimensional manifolds toward compact one-dimensional filaments. The *D_2_h* case represents the terminal stage of this reduction, where the convective organization reaches a state of maximal spatial fragmentation but minimal topological complexity (closed loops being the simplest one-dimensional closed manifolds).

As a concluding remark for this section, it is also worth highlighting that the *D_2_h* symmetry plays a dual role: it constrains the admissible flow modes while simultaneously enabling multiplicity of equivalent attractor states. On one hand, the presence of three orthogonal mirror planes restricts the flow field to configurations compatible with these symmetries, excluding any single dominant roll that would violate them. On the other hand, the same symmetry allows multiple equivalent vortical structures to coexist, each occupying a distinct spatial quadrant or octant. This degeneracy gives rise to a multitude of potential attractor locations, leading to the observed kaleidoscopic diversity of filamentary loops.

## 6. Conclusions

The results obtained in this study confirm that the adoption of thermally encoded boundary conditions consistent with the *D_2_h* (*mmm*) symmetry group fundamentally transforms the organization, morphology, and topology of vibrationally induced particle attractors in non-isothermal fluid-filled cavities. By enforcing such a highly constrained thermal architecture, featuring six opposite walls arranged to satisfy three mutually orthogonal mirror planes and twofold rotational symmetry about each principal axis, the system enters a qualitatively new dynamical regime essentially characterized (for relatively high values of the Gershuni number) by filamentary accumulation structures, which represent one-dimensional manifolds embedded within the three-dimensional flow domain.

The spatial topology of the overarching attractors is closed, and this is clearly reflected by the emergence of circular or elliptical particle loops/rings without free ends. Although their geometry is reminiscent of vortex lines or particle chains, their origin lies not in vorticity concentration but in the interplay between periodic thermovibrational modulation of particle paths, related inertial effects and particle–wall interaction. Remarkably, despite the intrinsically alternating (oscillatory) nature of the thermovibrational flow and the forcing itself (the imposed vibrations), these closed chains of particles are dynamically stable. The thickness of each chain is minimal, indicating strong local confinement and low transverse diffusion; nevertheless, such structures survive over long time intervals even as the entire pattern undergoes gentle, periodic oscillations synchronized with the external vibrational forcing. As an additional typical feature, the particle circuits occupy restricted planes, often aligned with the symmetry planes or solid boundaries of the cavity or slightly displaced depending on the strength and eccentricity of the surrounding rolls.

Notably, in many cases, multiple loops coexist and sometimes share common segments. These interconnections occur preferentially along the intersection lines of symmetry planes, and the resulting configurations often form interesting braided or lattice-like structures, suggestive of a higher-order organization emerging from the underlying flow network. Accordingly, a direct connection can be identified between the number and distribution of convective rolls and the topological dimensionality of the resulting particle formations.

In particular, comparison with earlier findings leads to the conclusion that, while in simpler systems dominated by a small number of rolls, the flow exhibits global coherence and supports the formation of extended accumulation surfaces; as additional thermal inhomogeneities are introduced, the convective field becomes increasingly more complex, and the effective dimensionality of the attractors decreases accordingly. Specifically, the *D_2_h* configuration, where the number of rolls reaches its maximum due to the multiplicity of symmetry-related thermal regions, represents a condition of pronounced flow-field fragmentation. For relatively high values of the Gershuni number, this flow field contains both instantaneous vortices, continuously alternating between clockwise and counterclockwise orientations, and time-averaged vortices, which are steady in nature and typically exhibit unequal strengths and eccentric positions when the vibrations are not perfectly perpendicular to the cavity walls.

These vortices and their interaction represent the constitutive elements of the “machinery” (the “collective” mechanism) leading to one-dimensional attractors. Specifically, while the particle structures owe their existence to the interplay between the instantaneous flow and inertial effects, time-averaged effects (flow) essentially contribute to their planar (compressed) appearance; i.e., they oppose particle transverse diffusion. The filamentary topology of the particle structures thus emerges as a natural consequence of the hierarchical fragmentation of the convective field and the interplay between instantaneous and average mechanisms. Such a combination can even result, in specific cases, in values of the parameter *K* (degree of accumulation) exceeding (for the same magnitude of the temperature difference and intensity of vibrations) the maximum value potentially achievable in the standard configuration with a uni-directional temperature gradient and uniformly heated or cooled walls. Moreover, in many cases, the eccentric positioning of the time-averaged rolls within the cavity enhances the spatial heterogeneity of the velocity field; that is, each roll contributes differently to the local velocity gradients, producing nonuniform zones of compression and expansion that further promote filament thinning.

It is clear that, beyond the fundamental interest that these phenomena attract because of their fascinating nature and the related theoretical link with the field that studies the dynamics of non-linear systems, the observed transition from 3D accumulation surfaces to filaments under increasing thermal complexity also carries notable technological implications. Such phenomena not only deepen our understanding of pattern formation and symmetry-breaking mechanisms in thermally forced environments but also suggest concrete strategies for manipulating dispersed phases in practical settings. In particular, the observed evolution of particle arrangements indicates that, by tailoring the spatial distribution of temperature on container walls, it may be possible to engineer flow fields that organize particles into predetermined patterns, a concept that clearly finds application in materials processing, crystal growth, or microfluidic manipulation in the energy field. These considerations thus justify future research in which the extensive numerical data generated by systematically varying problem parameters, thermal boundary conditions, and vibration direction will be used to train artificial intelligence algorithms capable of solving the associated inverse problem, that is, determining the optimal conditions required to produce particle structures with prescribed shape, multiplicity, and size.

## Data Availability

The data that support the findings of this study are openly available in the PURE repository of the University of Strathclyde under the record entitled “Data for Thermovibrationally-driven Ring-shaped Particle Accumula-tions in Corner-Heated Cavities with the *D_2_h* symmetry”.
